# Review of applications of CRISPR-Cas9 gene-editing technology in cancer research

**DOI:** 10.1186/s12575-021-00151-x

**Published:** 2021-07-15

**Authors:** Ziyi Zhao, Chenxi Li, Fei Tong, Jingkuang Deng, Guofu Huang, Yi Sang

**Affiliations:** 1 The Third Affiliated Hospital of Nanchang University, Nanchang, 330008 China; 2 Orthodontic Department of Affiliated Stomatological Hospital of Nanchang University, Nanchang, 330008 China

**Keywords:** CRISPR, Cas9, Cancer, High throughput screening, Cancer treatment

## Abstract

Characterized by multiple complex mutations, including activation by oncogenes and inhibition by tumor suppressors, cancer is one of the leading causes of death. Application of CRISPR-Cas9 gene-editing technology in cancer research has aroused great interest, promoting the exploration of the molecular mechanism of cancer progression and development of precise therapy. CRISPR-Cas9 gene-editing technology provides a solid basis for identifying driver and passenger mutations in cancer genomes, which is of great value in genetic screening and for developing cancer models and treatments. This article reviews the current applications of CRISPR-Cas9 gene-editing technology in various cancer studies, the challenges faced, and the existing solutions, highlighting the potential of this technology for cancer treatment.

## Introduction

Cancer is a unique genetic disease requiring multiple mutations [1], which promote cell proliferation, inhibit cell apoptosis, and alter the expression of specific antigens on the cell surface to evade recognition by the immune system through the activation/deactivation of multiple signaling pathways. Cancer cells are often invasive, wreak havoc on normal human tissue and cause distant metastasis through lymphatics and blood vessels. While notable progress has been made in cancer treatment (e.g., surgery, chemotherapy, radiation therapy, molecular targeted therapy, combined therapies, etc.), the incidences of side effects, recurrence, and chemotherapy/radiotherapy resistance are still high [2]. Therefore, exploring novel cancer-specific vulnerabilities is urgent. However, while the number of discovered driving mutations in cancer is still limited, new tools are needed to identify functional cancer driver genes.

As one of the most potent gene-editing tools to date, clustered regularly interspaced short palindromic repeats (CRISPR)-CRISPR-associated protein 9 (Cas9) technology has demonstrated applicability, simplicity, and efficient gene-editing capability compared to previously developed gene manipulation tools [3, 4]. At present, in cancer research, the applications of CRISPR-Cas9 mainly involve the screening of oncogenic mutations and tumor suppressors, the construction of in vivo and in vitro cancer models, and cancer gene therapy [5, 6]. With a relatively high editing efficiency and few off-target effects, the CRISPR-Cas gene-editing system is able to change the biological behavior of tumor cells from the level of the genome, reduce the destruction of normal human tissue cells, and increase the survival time of patients.

In this review, we first describe the CRISPR-Cas9 system as a versatile tool for genome engineering, and then, we outline various applications of the CRISPR-Cas9 system in functional gene screening. In addition to summarizing the CRISPR-Cas9 delivery system, multiple in vivo and in vitro models of different cancer types engineered by CRISPR-Cas9 are reviewed. Furthermore, the applications of CRISPR-Cas9 in cancer therapy are summarized. Finally, the limitations of CRISPR-Cas9 in practical applications and solutions to these limitations are discussed.

## Property and priority of CRISPR -Cas9 as an advanced gene-editing tool

To achieve efficient and accurate gene editing, a variety of techniques were developed, including RNA interference (RNAi), zinc finger nucleases (ZFNs), or transcription activator-like effector nucleases (TALENs). Initially discovered in *Caenorhabditis elegans*, RNAi is triggered by double-stranded RNA (dsRNA) [7]. When exogenous dsRNA is introduced into cells, it is first recognized and processed into 21–23 base pairs of small interfering RNAs (siRNAs) by the RNase III family ribonuclease Dicer. These siRNAs then mature and direct the RNA-induced silencing complex (RISC) to the target RNA, which leads to the ultimate destruction of the target RNA and gene silencing [8]. However, the exploration of specific genes through RNAi is limited, as RNAi impairs the expression of particular genes at the transcription level.

ZFNs and TALENs are engineered nucleases fused to sequence-specific DNA binding domains and nonspecific DNA cleavage modules. These engineered nucleases induce targeted DNA double-strand breaks (DSBs), which stimulate error-prone nonhomologous end-joining (NHEJ) or homology-directed repair (HDR) [9, 10]. Therefore, ZFNs/TALENs make genome editing possible. However, under the current status of the technology, the off-target activity of ZFNs is still high. Furthermore, TALENS may affect the chromatin microenvironment, and their binding activity may be impaired in regions of condensed chromatin [11].

**Fig. 1:**
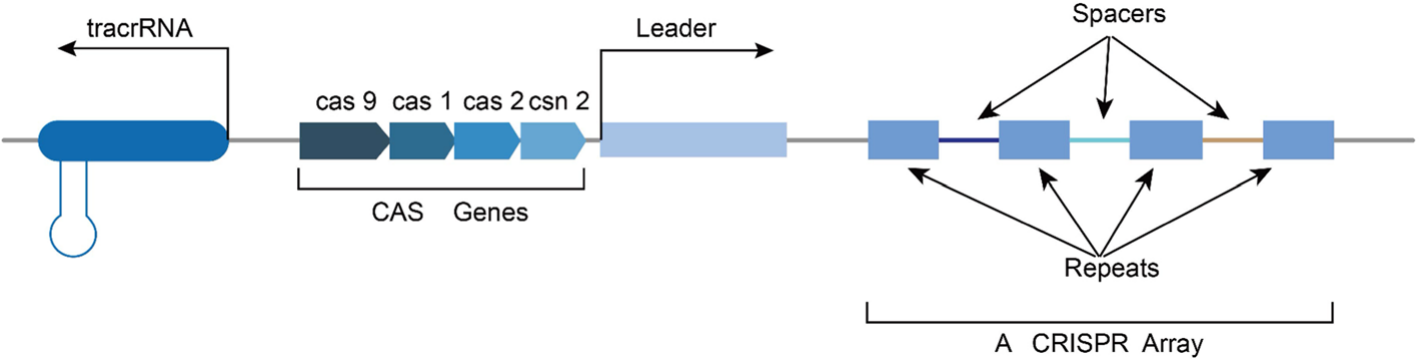
Genomic structure of the bacterial CRISPR-Cas9 system: CRISPR is a special repetitive sequence-spacing array of clustered bacterial genomes made up of spacing sequences from phage DNA and recombination sequences from the host bacteria genome adjacent to the Cas gene. CRISPR mainly includes tracrRNA genes, Cas genes, leaders, repeat sequences and interval sequences

First discovered in *Escherichia coli* (Fig. 1), the CRISPR-Cas system is an adaptive immune system that defends against foreign invasive nucleic acids [12]. The “classical” CRISPR-Cas system family mainly consists of 6 types that are grouped into two classes. Class I members (type I, III, and IV systems) employ multiple Cas proteins, while Class II members (type II, V, and VI systems) have only one Cas protein with multiple domains [13]. Therefore, Class II CRISPR-Cas systems are easier to use in genetic engineering applications. Among the type II CRISPR Cas system members, CRISPR-Cas9 developed from *Streptococcus pyogenes* (e.g., “*S. pyogenes*”) is the most extensively applied system in mammals [14].

**Fig. 2:**
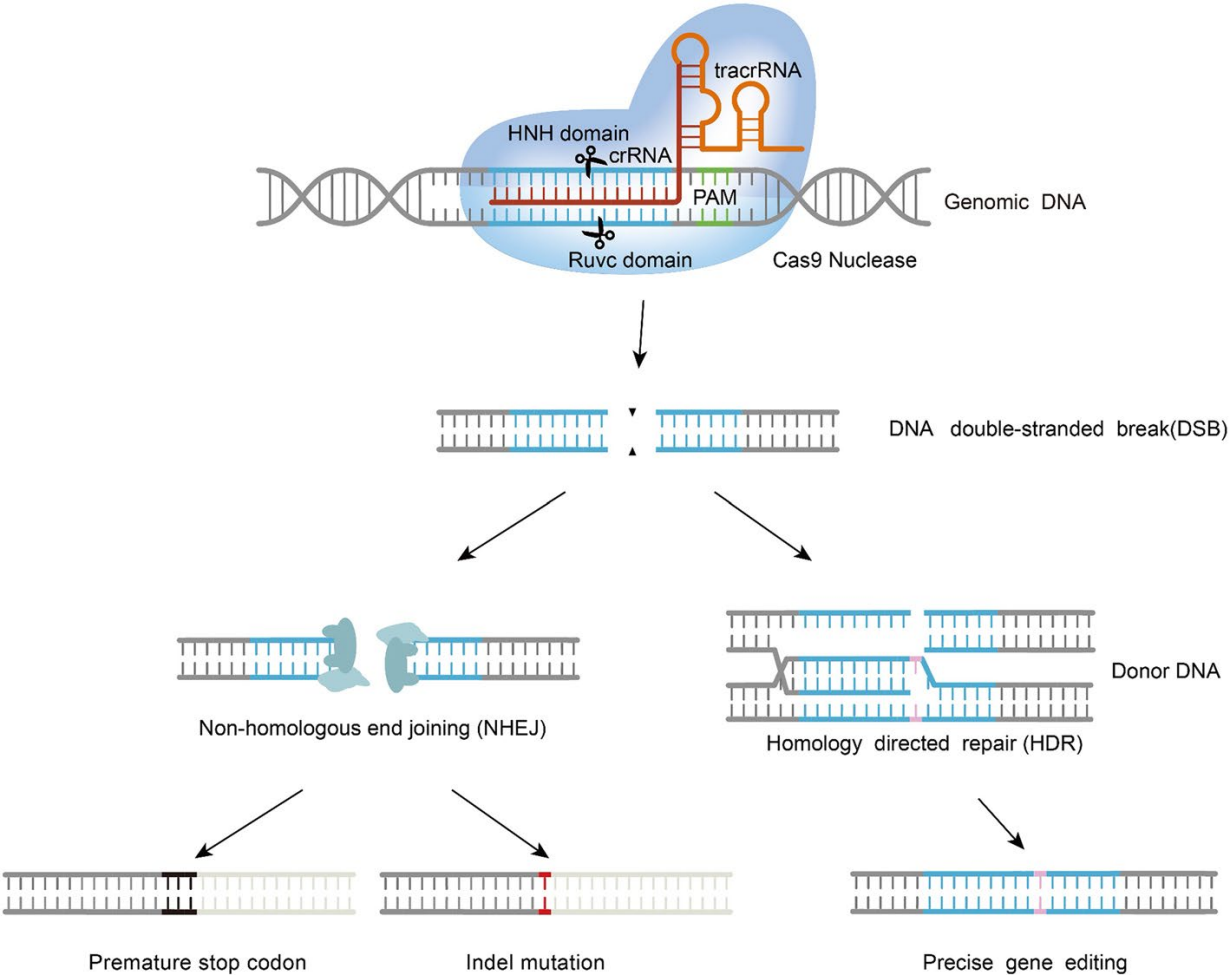
The molecular mechanism of CRISPR-Cas9: SgRNA consists of tracrRNA and crRNA. The crRNA contains a 20-base recognition sequence and an additional sequence that complements tracrRNA. TracrRNA is hybridized with crRNA and combined with the Cas9 protein to form the CRISPR-Cas9/sgRNA complex, which produces DSBs at the target sites of the genome. SgRNA is often designed to contain two key fragments: a double-stranded RNA structure that binds Cas9 at the 3'-end and a guide sequence that binds the target DNA sequence at the 5'-end. SgRNA can recognize a specific sequence in the genome. The Cas9 protein is an endonuclease containing two domains (RuvC and HNH). The RuvC domain cleaves the noncomplementary DNA strand, while the HNH domain cleaves the complementary DNA strand. After DSBs are formed, either the NHEJ pathway or HDR pathway is activated. The NHEJ pathway often leads to insertions/deletions (Indel) and an open reading frame shift of the target gene. In contrast, the HDR pathway requires a donor DNA template to repair DSBs. The donor DNA template is used to insert the correct DNA sequence precisely into the target site

A typical CRISPR-Cas9 system is composed of a single guide RNA (sgRNA) and Cas9. SgRNA is a synthetic fusion between CRISPR RNA (crRNA) and trans-activating crRNA (tracrRNA). With a recognition sequence of approximately 20 bases, crRNA specifically binds to the target site while the additional sequence complements the tracrRNA portion [14, 15]. The Cas9 protein is an endonuclease with an Ruvc domain and HNH domain. The HNH domain cleaves single DNA strands complementary to the sgRNA, while the Ruvc domain cleaves noncomplementary DNA strands [16]. During editing, the 5'-end of sgRNA explicitly recognizes the target site, and the 3'-end binds the Cas9 protein, guiding it to the target sequence. The sgRNA/Cas9 complex recognizes the protospacer-adjacent motif (PAM) and cleaves double-stranded DNA [17]. Adjacent to the target DNA sequence, PAM is usually composed of NGG or NAG (N = A, T, G, or C)in the CRISPR Cas system developed from *S. pyogenes* [18]. When DSBs are induced, the HDR pathway is activated by the provided template DNA to achieve gene knock-in or base pair replacement. Alternatively, the error-prone NHEJ pathway may be activated (Fig. 2). Specific gene knockout is achieved since NHEJ repair often induces insertion/deletion (Indel) mutations, leading to an open reading frame shift of the target gene [3].

## Application of CRISPR -Cas9 in gene screening

**Fig. 3:**
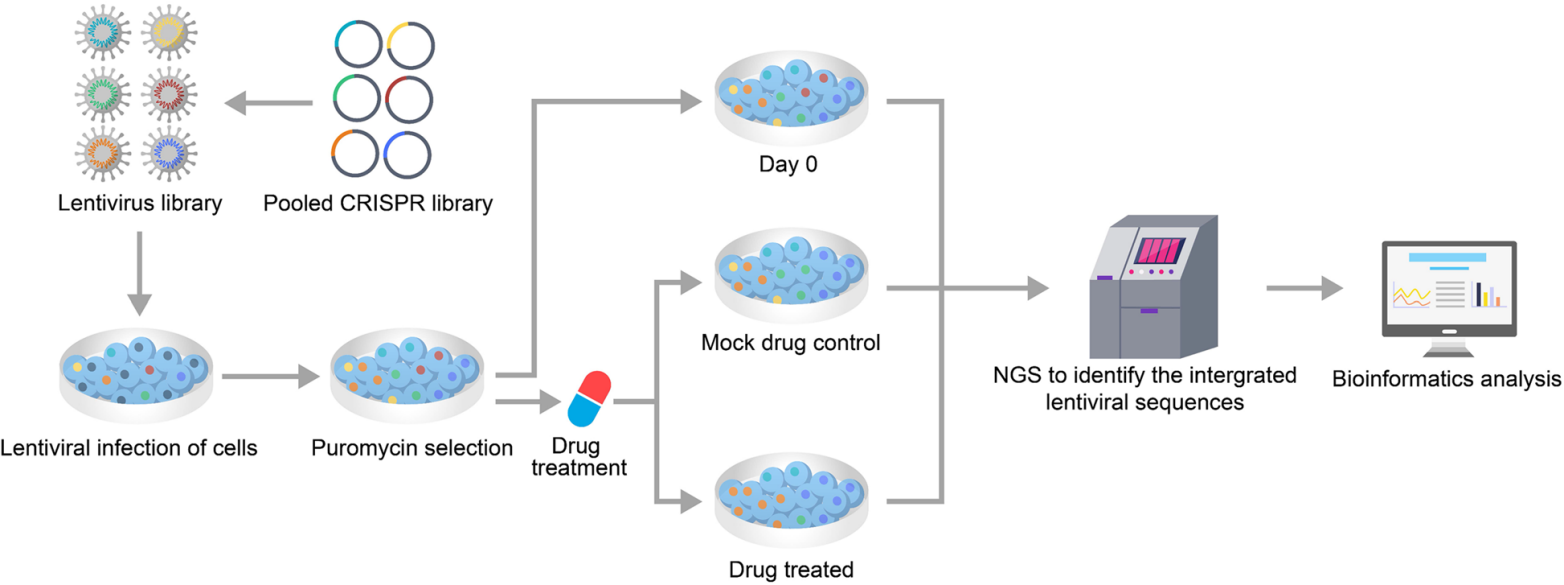
Basic steps of CRISPR-Cas9 gene screening: First, plasmids were constructed that expressed the corresponding CRISPR components, they were packaged as viruses, libraries were built, and the cancer cells to be studied were infected. Then, uninfected cancer cells were removed by drug selection (e.g., puromycin), and the screened cancer cells were divided into three groups: the day 0 population, drug-treated population (treatment) and control population (mock-drug control, typically treated with a vehicle such as DMSO). After specific drug treatments, genomic DNA was extracted from the transduced cells, and high-throughput sequencing was performed to sequence the sgRNA coding region that the virus had integrated into the host genome. Finally, bioinformatics analysis was performed to compare the sequencing results of the three groups of cancer cells, and the genes that were affected by specific drugs were preliminarily obtained

To understand the molecular mechanisms of tumorigenesis and explore novel therapeutic targets, it is essential to identify the core genetic dependencies and key oncogenic mutations in cancers (Fig. 3). Therefore, efficient and unbiased in vitro and in vivo gene screening is imperative.

RNAi techniques are applied in gene screens of siRNA/shRNA libraries. However, while RNAi targets mRNA transcripts and gene-specific knockout is usually partial or incomplete, the scope of gene screening by RNAi is limited [19]. CRISPR-Cas technology significantly overcomes this limitation, as the application of CRISPR-Cas9 in gene screening offers the possibility of targeting a more comprehensive range of the cancer genome [20, 21]. It has been proven that the proliferation of multiple cancer cell lines depends on maternal embryonic leucine zipper kinase (MELK) through RNAi or small-molecule targeted drugs. Hence, OTS167 is considered a new chemotherapy drug targets MELK [22–24]. However, Ann Lin et al. found no significant change in the fitness of breast cancer cell lines or six other cancer cell lines after MELK was mutated by CRISPR-Cas9. Furthermore, OTS167 still repressed the proliferation of MELK-knockout cell lines. Therefore, the authors suggested that OTS167 might impede cell division by other mechanisms [25]. This study highlights the need to screen and validate drug targets in preclinical studies using the CRISPR-Cas9 technique. This study also reveals the challenges of using CRISPR technology to correctly identify essential genes for cancer. On the other hand, Michiko Kodama et al. combined a shRNA library with a genome-wide CRISPR-Cas9 library to perform in vivo loss-of-function screening, which identified KPNB1 as a new therapeutic target for epithelial ovarian cancer. Interestingly, more gene deletions were observed in tumors using CRISPR-Cas9 than in tumors using shRNA, showing that functional differences exist between the two screening methods to some extent [26].

Genome-wide CRISPR-Cas9 screening has produced numerous high-quality data [27–29]. Sidi Chen et al. obtained specific functionally defective mutations essential for tumor growth and metastasis using genome-wide CRISPR screening, such as *Cdkn2a, Fga,* and *Cryba4 *[30]. Similarly, Ryan d. Chow et al. identified several functional suppressors and the cooccurrence and correlation of specific mutations in glioblastoma through in vivo CRISPR screening. They identified cooccurring driver combinations including *B2m-Nf1*, *Mll3-Nf1* and *Zc3h13-Rb1* by comutation analysis [31]. CRISPR-Cas9 technology also provides a new approach for functional oncogene fusion screening. Gabriele Picco et al. concluded that most gene fusion events did not affect tumor fitness according to functional fusion analysis using CRISPR-Cas9 loss-of-fitness data [20].

CRISPR-induced screening strategies typically target exon mutations at the 5’-end of candidate genes [21, 32, 33]. Junwei Shi et al. proposed that targeting exons encoding functional protein domains with CRISPR-Cas9 enables a higher proportion of deletion mutations [34]. On the other hand, to improve screening efficiency, Najm FJ et al. paired *Staphylococcus aureus* Cas9 (SaCas9) with SpCas9 and established design rules for SaCas9 sgRNA using a machine learning method to achieve dual targeting in multiple types of cells [35]. These studies promote the use of a gene screening strategy with the CRISPR-Cas9 system. However, there is still an undeniable risk of bias. Genomic targeting by CRISPR-Cas9 induces a gene-independent antiproliferating cell response, which may bias the screening of critical genes in the amplified region [36].

In most cases, the efficacy of anticancer drugs decreases gradually in the clinic, which is mainly related to the upregulated expression of drug-resistant genes in tumor cells. Therefore, drug-resistant gene screening is of great significance for improving the clinical efficacy of drugs and proposing new combined treatment schemes. TP53 and SOCS6 are imatinib-resistant candidate genes in gastrointestinal stromal tumors (GISTs) that were identified using the genome-wide CRISPR-Cas9 knockout screening technique. It is also suggested that DBP, NR3C1, TCF12, and others are involved in the imatinib-resistant mechanism of GIST, which provides a direction for exploring new therapeutic targets and further elucidating the mechanism of imatinib resistance [37]. Wei L et al. identified the first activated enzyme phosphoglycerate dehydrogenase (PHGDH) in the serine synthesis pathway (SSP) as a critical driver of sorafenib resistance through a genome-wide CRISPR-Cas9 screening and verified that the PHGDH inhibitor NCT-503 was synergistic with sorafenib to impair hepatocellular carcinoma (HCC) growth in vivo [38]. This study also showed the great potential of CRISPR-Cas9 for identifying drug-resistant genes.

Although the CRISPR/Cas9 system has received a large amount of attention, DSBs caused by this system frequently result in undesirable insertions, deletions, and rearrangements [39, 40]. To address this problem, researchers have identified nuclease-dead mutants of Cas9 (dCas9) that abolish cleavage but do not impair DNA binding [16]. dCas9 can block the transcription of some genes in bacteria and mammalian cells. Gilbert et al. showed that when catalytically dead Cas9 lacking endonuclease activity was coexpressed with sgRNA, a DNA recognition complex was generated that could specifically interfere with transcriptional elongation, RNA polymerase binding, or transcription factor binding. This process is called CRISPR interference (CRISPRi) [41]. CRISPRi technology has enabled genome-scale screenings of gene function in mammalian cells, which have been valuable for the identification of genes in noncoding and coding regions. S John Liu et al. used CRISPRi to screen 5689 lncRNA loci in human glioblastoma (GBM) cells, identifying 467 hits that modified cell growth in the presence of clinically relevant doses of fractionated radiation. They identified lncGRS-1 as a glioma-specific therapeutic target and established a generalizable approach to rapidly identify novel therapeutic targets in the large noncoding regions of the genome to enhance radiation therapy [42]. Similarly, Shiyang Liu et al. designed a CRISPRi sgRNA library to target the transcription start site (TSS) of each of the Wnt-regulated lncRNAs. They selected a total of 1503 Wnt-regulated lncRNAs for a CRISPRi screen of the HPAF-II cell line (a human pancreatic adenocarcinoma cell), which contained 3151 transcripts including different isoforms. The researchers found that the four Wnt-regulated lncRNA loci (RP11-481J2.3, LINC00910, LINC00263, CCAT1) that were hits in the in vivo and in vitro screenings were essential for the growth of HPAF-II cancer cells [43]. dCas9 can also be used to activate gene expression, in an approach termed CRISPR activation (CRISPRa). Bikard et al. showed that a fusion between the omega subunit of the RNA polymerase and a Cas9 nuclease mutant directed to bind upstream promoter regions could achieve programmable transcription activation in *Escherichia coli *[44]. In eukaryotic cells, researchers fused 4 copies of the well-characterized transcription activator VP16 or a single copy of the p65 activation domain (AD) to dCas9. They cotransfected dCas9-VP64 or dCas9-p65AD and a sgRNA construct that targeted the Gal4 UAS (upstream activation sequence) into a HEK293 reporter cell line expressing a Gal4 UAS-GFP reporter. The results revealed that both dCas9-VP64 and dCas9-p65AD could effectively activate reporter gene expression [45]. Cytarabine (1-p-d-arabinofuranosylcytosine, Ara-C) is a front-line chemotherapy agent frequently used in the treatment of acute myelocytic leukemia(AML) [46]. However, approximately 30% to 50% of patients relapse with chemotherapy-resistant disease. Assaf C Bester et al. carried out functional screening with CRISPRa based technologies using a new genome wide non-coding sgRNA (CRISPR activation of lncRNA, CaLR) library. They identified candidate lncRNAs that facilitated resistance to Ara-C treatment by the CaLR approach, such as GAS6-AS2 and AC008073.2 [47]. The strong performance of CRISPRi and CRISPRa in genetic screenings is remarkable. Over the next few years, improvements in the design of sgRNAs and dCas9 constructs for CRISPRi and CRISPRa may further enhance the utility of these approaches.

## The CRISPR -Cas9 delivery system used in cancer research

Several delivery systems have been developed to efficiently deliver CRISPR components to target cells, which are mainly divided into viral-based delivery systems and nonviral delivery systems.

### Viral-based delivery system

Adeno-associated virus (AAV), lentivirus, and adenovirus are commonly used to deliver the plasmid-based CRISPR-Cas9 system in cancer research. As nonpathogenic adeno-associated virus infects both dividing and nondividing cells with mild immunogenicity [48, 49], Wang G et al. successfully induced liver cancer by mutating the liver of Cre-inducible Cas9 mice with an adeno-associated virus as a carrier to deliver the sgRNA library aimed at possible tumor suppressor genes, such as *Trp53*, *Setd2*, *Cic*, and *Pik3r1 *[50]. With relatively lower immunogenicity and a higher infection efficiency, the expression of the genes transfected by lentivirus lasts longer [3]. After genome-wide CRISPR-Cas9 screening with lentiviral vectors, it was demonstrated that RNASEH2 deficiency could be induced by a synthetic lethal agent with inhibition of ATR in vitro and in vivo [51]. However, the packaging capacity of adeno-associated viruses and lentiviruses was limited [52, 53]. Therefore, genetically engineered mice expressing Cas9, which can be edited by simply introducing sgRNA, were constructed to overcome the packaging limitation. Randall J. Platt et al. established a Cre-dependent Cas9 gene knockin mouse. They used AAV vectors to introduce *p53*, *Lkb1*, and *Kras* mutations into the lung, leading to the formation of lung tumors [54]. Unlike AAVs and lentiviruses, adenoviruses have larger transgene sizes and fewer sequence restrictions [55]. The CRISPR component was also delivered to mouse cells through tracheal recombinant adenovirus to induce *Eml4*-*Alk* gene fusion, which induced lung tumorigenesis with high penetrability and low latency [56]. However, the high immunogenicity and limited tissue orientation restrict the application of adenovirus vectors [57].

### Nonviral delivery systems

Although the delivery efficiency of nonviral delivery systems is relatively lower than that of virus vectors, these systems are much safer for the host. Hydrodynamic injection, electroporation, nanoparticles, and transposon carriers are standard nonviral delivery methods. Dr. Zeming Chen et al. developed liposome-templated hydrogel nanoparticles (LHNPs) combined with minicircle DNA technology to deliver a CRISPR-Cas9 system targeting Plk1 (polo-like kinase 1), which effectively repressed tumor growth and improved the survival rate of tumor-bearing mice [58]. As an alternative carrier to deliver guide RNA (gRNA) libraries for in vivo screening, *piggyBac* (PB) transposons can achieve transduction efficiencies of 86.85% to 92.43% in mouse livers [59]. However, nonviral delivery system often fail to achieve tissue specificity, which may be able to be overcome by appropriate modification of the Cas9 protein. Asialoglycoprotein receptor (ASGPr) is a c-type lectin expressed almost exclusively on the surface of liver cells. Romain Rouet et al. designed a Cas9 protein that carried the ASGPr ligand and formed a complex with sgRNA to deliver the CRISPR-Cas9 system specifically to liver cells [60].

## Application of CRISPR -Cas9 in cancer models

Cancer involves multiple point mutations, translocations, and chromosomal losses and gains, which complicate the cancer genome [61]. Therefore, it is challenging to identify driver mutations in cancer. It is necessary to model the development and progression of cancer and develop cancer models with human disease characteristics. CRISPR-Cas9 accelerates this process due to its efficient and straightforward gene-editing capability. Cancer models can be roughly divided into two categories: in vitro and in vivo. Common in vitro models are organoids—which are tiny, self-organized three-dimensional tissue cultures derived from stem cells. A cancer model with similar biological behavior to that of a tumor can be constructed by knocking out specific genes using CRISPR-Cas9 technology to simulate the mutation spectrum observed during the occurrence and development of cancer. Commonly applied in mice, in vivo cancer models are constructed through the induction of oncogenic mutations or chromosomal rearrangements by delivering the CRISPR-Cas9 system to specific organs or tissues in mice.

### Hepatocellular carcinoma

To model liver cancer, it is common to inject CRISPR components through the tail vein in mice. For example, Wen Xue et al. constructed a mouse liver cancer model by targeting the tumor suppressor genes *Pten* and *p53* in the mouse liver [62]. Furthermore, to accurately mimic the characteristics of complex mutations in cancer, researchers are exploring the possibility of triggering multiple mutations in mouse liver cells using CRISPR-Cas9. Julia Weber et al. established a mouse liver cancer model by introducing multiple tumor suppressor gene mutations in the mouse liver through the CRISPR-Cas9 system. They chose to target *Trp53*, *Smad4*, *Pten*, *Cdkn2a*, *Apc*, *Arid1a*, *Tet2*, *Brca1/2* and others*.* In defined genetic (KrasG12D) and liver damage models (CCl4), the researchers showed for the first time that CRISPR/Cas9 somatic gene targeting can be used to induce HCC. After functional genomics analysis, they confirmed the role of chromatin modifiers in liver tumorigenesis [63].

### Pancreatic cancer

Pancreatic ductal adenocarcinoma (PDAC) is a highly malignant cancer with a poor prognosis. According to related research, pancreatic cancer is expected to be the second leading cause of cancer-related death by 2030 [64]. To understand the underlying molecular processes of pancreatic cancer, various pancreatic cancer models have been constructed. Roman Maresch et al. successfully simulated the formation of pancreatic cancer by inducing multiple gene mutations (such as *Trp53*, *Smad4*, *Pten*, *Apc* and others) in the mouse pancreas through a plasmid-based CRISPR-Cas9 system by electroporation. They also used CRISPR-Cas9 multiplexing for negative selection screening of the pancreas and confirmed *Brca2* inactivation as a vulnerability of pancreatic cells with *Kras* mutations [65]. Furthermore, Shin-Heng Chiou et al. generated single insertion transgenic mice through site-directed integration of an *LSL*-Cas9 cassette into the *H11* locus (*H11*^LSL−Cas9^). They established a mouse pancreatic cancer model by injecting an adenovirus vector directly into the pancreas of transgenic mice through a retrograde pancreatic catheter injection. Rapid tumor growth was then obtained by targeting *Lkb1* using CRISPR-Cas9 [66].

### Colorectal cancer

Colorectal cancer (CRC) is one of the most common malignancies in the United States [67]. Organoid techniques can be applied in combination with the CRISPR-Cas9. As mutations driven by replication errors accumulate in *MLH1*-deficient colorectal cancer cells, the profile observed in mismatch repair-deficient colorectal cancers can be accurately modeled by knocking out *MLH1* using the CRISPR-Cas9 technique [68]. In vivo models can be established by transplanting cells derived from colorectal cancer patients into the ventral and renal capsules and spleen of mice [69, 70]. However, this method leads to the formation of tumors in ectopic sites. Jatin Roper et al. established a mouse model of colorectal cancer by inducing tumor formation in the distal colon of mice by a colonoscopy-guided mucosal injection of CRISPR-Cas9 components containing lentiviral constructs. The researchers showed that in situ gene editing was successful in wild-type mice using lentiviral constructs expressing a short guide RNA targeting *Apc* and Cas9 to model CRC. With the introduction of colonoscopy, site-directed tumors can be rapidly and efficiently induced in mouse models [71].

### Lung cancer

Lung cancer is the most common cause of cancer deaths in men and women worldwide [72]. Lung tumors with histopathological and molecular characteristics of human lung cancer can be induced in mice through CRISPR-Cas9-mediated gene mutations and chromosome rearrangement. Platt RJ et al. used the Cas9 gene to deliver multiple sgRNAs through an AAV vector to mice, causing functional deletion mutations of *p53* and *Lkb1* in the lung, as well as mutations of Kras G12D mediated by homologous directed repair, and established a mouse lung adenoma model [54]. On the other hand, Danilo Maddalo et al. induced chromosomal rearrangements using the virus-mediated CRISPR-Cas9 system to express the *Eml4*-*Alk* fusion gene, generating a mouse model of Eml4-Alk-driven lung cancer that responded to treatment with ALK –inhibitors [56].

### Breast cancer

Breast cancer is the most common cancer among American women (excluding skin cancer) and the second leading cause of cancer-related death in women after lung cancer [73]. A variety of breast cancer models have been developed using the CRISPR -Cas9 system. Annunziato S et al. produced knockout mice through conditional Cre expression in the cytosine base editor. After in situ delivery of the sgRNA encoding vector, efficient point mutations could be induced in one or more endogenous genes(*Pik3ca, Akt1* and so on) by CRISPR-Cas9 technology, and the system was successfully applied to the triple-negative breast cancer model [74]. In addition to in situ induction, organoid transplants of breast cancer in mice can also mimic breast tumor production in humans. Dekkers JF and colleagues used CRISPR-Cas9 to target four breast cancer-related tumor suppressor genes (*P53, PTEN, RB1,* and *NF1*) in breast progenitor cells from six donors. After transplantation of the 1/6 *P53/PTEN/RB1* mutant cell line and 3/6 *P53/PTEN/RB1/NF1* mutant cell line into mice, long-term cultures were obtained, and ER + intracellular tumors were formed [75].

## Application of CRISPR -Cas9 in cancer treatment

**Fig. 4:**
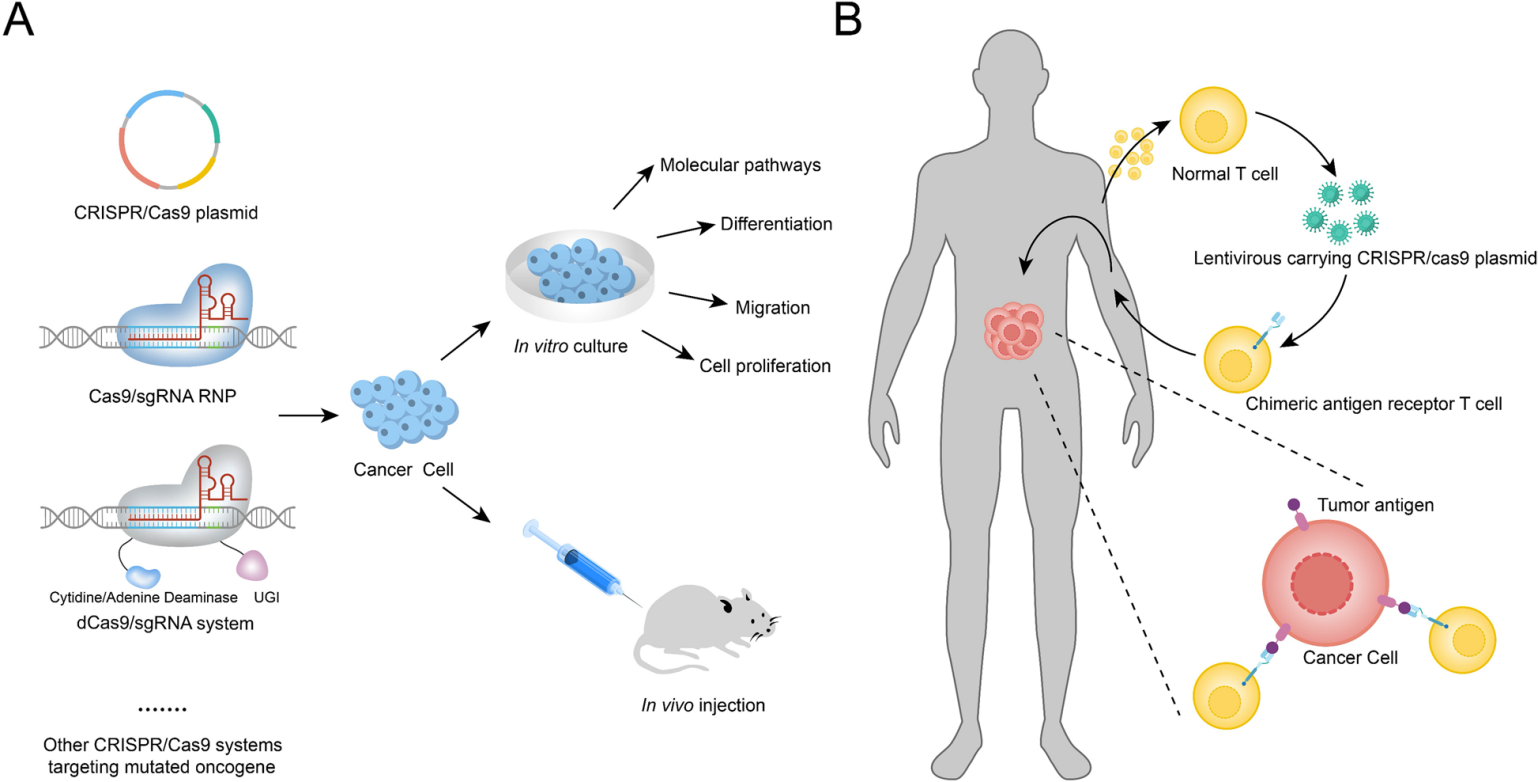
Applications of CRISPR-Cas9 technology in cancer research: (**A**) Modifications of cancer cell genomes with different types of CRISPR-Cas9 systems in vitro and in vivo; (**B**) CAR T cell therapy combined with CRISPR-Cas9 technology

Researchers are actively exploring the potential of CRISPR-Cas9 for cancer treatment by targeting specific sequences in the cancer genome or driver mutations associated with cancer progression (Fig. 4). It is common to induce apoptosis of tumor cells with CRISPR-Cas9 targeting oncogenic mutations. Koo T et al. used the CRISPR-Cas9 technique to distinguish oncogenic mutants from wild-type EGFR alleles and then eliminated oncogenic multiactive EGFR alleles with high accuracy, which significantly reduced tumor size in human lung cancer xenograft mouse models [76]. In addition, Chen ZH et al. identified the unique sequence produced by TMEM135-CCDC67 or MAN2A1-FER gene rearrangement in the genome of cancer cells using the CRISPR-Cas9 technique and introduced the gene encoding herpes simplex virus type 1 thymidine kinase (HSV1-tk). Under ganciclovir treatment, HSV1-tk initiated drug accumulation in cells, leading to cell apoptosis, while no such effect was observed in HSV1-tk-negative mammalian cells. A decreased tumor load in mouse xenograft models was observed in the HSV1-tk-introduced group, and no mice died during the study [77]. These strategies emphasize the ability to selectively kill cancer cells without any adverse effects on normal tissue cells, which is essential for clinical cancer treatment.

Nevertheless, it is still difficult to generate specific point mutations with the CRISPR-Cas9 system without producing any byproducts, such as insertions, deletions, and rearrangements [39, 40, 78, 79]. To accurately generate point mutations, a cytosine base editor (CBE) that enables the conversion of C-G to A-T base pairs without DSBs has been developed [80, 81]. Based on the CRISPR-Cas9 system, dCas9, cytidine deaminase, and uracil DNA glycosylase inhibitor (UGI) were fused. First, gRNA directs the dCas9 fusion to bind to the specific site without causing DSBs. Then cytosine deaminase deaminates cytosine, transforming C into U and forming a U-G intermediate. At the same time, UGI inhibits uracil n-glycosylase (UNG), protecting the U-G intermediates from being converted back to C-G. Finally, U-G is converted to A-T through the DNA replication process. Liu et al. corrected the dominant-negative p53 mutation Tyr163Cys using CBE in human breast cancer cell lines (HCC1954 cells) with a deletion formation rate ≤ 0.7%, which provides an efficient and accurate approach to cancer treatment [81].

With the in-depth understanding of tumor immunology, tumor immunotherapy has become a research hotspot. Programmed cell death protein 1 (PD-1) checkpoint blockade immunotherapy and chimeric antigen receptor (CAR) T-cell therapy are relatively mature tumor immunotherapy options. Immunotherapy with PD-1 checkpoint blockade has made significant progress in treating lung cancer and advanced hematologic malignancies [82, 83]. However, this therapy is not effective for patients with low programmed death ligand 1 (PD-L1) expression [84]. To enhance the efficacy of anti-PD-1 immunotherapy, new targets can be identified by CRISPR-Cas9 screening. Manguso RT et al. found that increased expression of Ptpn2 induced resistance to PD-1 checkpoint blockade in tumor cells through in vivo CRISPR screening and confirmed that knockout of Ptpn2 in tumor cells could increase the sensitivity of tumor cells to PD-1 checkpoint blockade [85]. CAR-T cell therapy is efficient for treating B cell acute lymphoblastic leukemia and chronic lymphoblastic leukemia but shows little antitumor activity against most solid tumors [86, 87]. To increase the effectiveness of CAR-T cell therapy, approaches using CRISPR-Cas9 technology have been proposed. For example, CAR-T cells can be modified genetically using CRISPR-Cas9 to increase their antitumor activity. Justin Eyquem et al. showed that directing a CD19-specific CAR to the T-cell receptor α constant (TRAC) locus using the CRISPR-Cas9 technique could not only induce uniform CAR expression in human peripheral blood T cells but also enhance T-cell potency. Furthermore, the edited cells performed much better than the conventionally generated CAR-T cells in the mouse model of acute lymphoblastic leukemia [88]. TCR, HLA class I molecule, and PD-1-deficient CAR-T cells have also been developed using the CRISPR-Cas9 system to enhance cancer cell killing while reducing allogeneic reactivity [89].

CRISPR-Cas9 approaches can target noncoding regions as well as coding regions of genes. Noncoding RNAs are a class of RNA molecules in the transcriptome that do not encode proteins and play essential roles in regulating gene expression and affecting the biological behavior of various cancers. A class of noncoding RNAs, long noncoding RNAs (lncRNAs), have been shown to affect cancer progression. Yu Duan et al. found that the lncRNA SNHG3 was significantly downregulated in thyroid papillary carcinoma (PTC) tissues and cell lines and that the expression of SNHG3 was negatively correlated with the TNM stage and poor prognosis in PTC patients. The removal of SNHG3 by CRISPR-Cas9 technology promoted the proliferation, migration, and invasion of PTC cells. Therefore SNHG3 might be a promising candidate target for PTC [90]. Similarly, Xin Wang et al. knocked out the endogenous long noncoding RNA RP11-159K7.2 in TU-212 cells and AMC-HN-8 cells with the CRISPR-Cas9 system. The results showed that RP11-159K7.2 knockout reduced the proliferation and invasion of laryngeal squamous carcinoma cells (LSCC) in vivo and in vitro, suggesting that lncRNA RP11-159K7.2 might be a potential biomarker for LSCC therapy [91]. On the other hand, microRNAs (miRNAs) are attracting increasing attention for their role in cancer progression. MiR-3064 has been reported to be an important tumor suppressor in ovarian cancer [92]. However, other research has reached a different conclusion. Lenti-CRISPR-miR-3064 vectors were transfected into PaCa-2 (pancreatic ductal adenocarcinoma cell line), which was injected subcutaneously into nude mice. In terms of the tumor growth rate and size, miR-3064 knockdown significantly inhibited the growth of PaCa-2 xenograft tumors in mice, indicating that miR-3064 might act as a tumor suppressor or oncogene in different human cancers and might represent a potential target for PC therapy [93].

## Limitations and future directions CRISPR-Cas9: Off-target effects and immune response

Although CRISPR-Cas9 technology has significant advantages regarding its gene-editing efficiency, targeted sequence range, and simplicity, it is undeniable that its off-target effects impede its clinical application. Jinek et al. first found that the seed sequence defined as 12 bases based on the 3'-end of the sgRNA sequence, adjacent to the upstream PAM, had a low tolerance for CRISPR-Cas9 base mismatches [16]. At the same time, Yanfang Fu et al. proposed that Cas9 endonuclease could tolerate mismatches of one or two bases and that the activity of the targeted gene was more sensitive to mismatches at the 3'-end of the sgRNA targeting sequence [94]. In addition to base mismatches, the deletion and insertion of base pairs into DNA sequences can also lead to off-target effects. Yanni Lin et al. found that the CRISPR-Cas9 system also cleaved genomic sites when DNA sequences contained insertions or deletions compared to RNA guide chains [95].

New techniques have been developed to sensitively detect off-target effects and identify new off-target sites. Shengdar Q. Tsai et al. described whole-genome unbiased-identification sequencing (GUIDE-seq), which relies on the capture of double-strand oligonucleotides to detect CRISPR-Cas-induced double-strand breaks in DNA, and found off-target sites that were not recognized by the existing calculation methods [78]. Nevertheless, GUIDE-seq requires individual transfection of each target or cell source and is not as sensitive as in vitro methods, making it difficult to scale [96]. To improve the detection sensitivity, circularization for in vitro reporting of cleavage effects by sequencing (CIRCLE-seq) was developed to identify off-target mutations associated with cell-type-specific single-nucleotide polymorphisms [97, 98]. However, the considerable workload of CIRCLE-seq and the multiple processing reactions required for a single analysis make it difficult to analyze a large number of targets in parallel [96].

On the other hand, circularization for high-throughput analysis of nuclease genome-wide effects by sequencing(CHANGE—seq), a scalable, automatic marking method based on the label, in vitro measurement of Cas9 genome-wide activity was developed. CHANGE-seq was applied to 110 single guide RNA targets at 13 therapeutic-related sites in human primary T cells and identified 201,934 off-target sites, enabling machine learning model training to predict off-target activity [96]. In addition to techniques for detecting off-target sites in vitro, researchers are also trying to detect off-target effects in vivo. Pinar Akcakaya and colleagues adopted Verification of In Vivo Off-targets (VIVO), which has divided into two steps. In the initial in vitro "discovery" step, CIRCLE-seq is used to identify a set of potential off-target cleavage sites of nucleases. In the "confirmation" step in the second individual, indel mutation detection was performed on the loci identified by CIRCLE-seq in the target tissue treated with the nuclease, which mainly allows in vivo detection of the off-target effects of genomic CRISPR-Cas nuclease [99]. These techniques will allow further evaluation of the safety of using CRISPR-Cas9 in gene therapy.

There are currently two main approaches to reduce off-target effects: 1) optimization of the design rules of sgRNA and 2) selection of Cas9 endonuclease variants. Doench JG et al. integrated mouse and human genome-wide libraries, analyzed the off-target activity of thousands of sgRNAs and optimized their genome-wide libraries based on their improved algorithms for predicting on- and off-target activities [100]. Meanwhile, Benjamin P. Kleinver and colleagues described a high-fidelity variant of SpCas9-HF1 with a similar targeting activity to that of most wild-type SpCas9s, which showed no detectable off-target effects [101].

Another obstacle to the application of CRISPR-Cas9 is that the host may have an immune response to the SpCas9 protein, limiting the applicability of CRISPR-Cas9 to in vivo experiments and therapeutic applications. Wang D et al. used an adenovirus (Ad) vector to deliver SpCas9 targeting *Pten* to the mouse liver, which led to an increase in the IgG content in mice, suggesting that SpCas9 may induce humoral immunity and a specific cellular immune response [102]. Similarly, Chew WL et al. found that expression of Cas9 in adult anterior tibial muscle via AAV delivery of CRISPR components resulted in enlarged draining lymph nodes and an increased cell count. The expression of Cas9 in muscle significantly increased the frequency of CD45 + hematopoietic cells, which were enriched around transgene-expressing myofibers [103].

To overcome the immunogenicity of SpCas9, Ajina R et al. crossed Rosa26-LSL-Cas9 knockin (Cas9-KI + / +) male mice in the C57BL/6 J background with WT C57BL/6 J female mice to obtain heterozygous Cas9 transgenic mice. Multicolor flow cytometry analysis found no significant difference in the spleen immune cell population between the original Cas9 transgenic mice and the original WT C57BL/6 J mice, providing a promising approach and idea for overcoming the immunogenicity of SpCas9 in in vivo applications [104].

## Conclusion

As a genetic disease characterized by malignant cell proliferation, cancer is a severe threat to human health and requires an effective way to determine its complex genetic dependence. CRISPR-Cas9 gene-editing technology has broad application prospects in cancer research and can lead to the development of effective treatment strategies and improve research. In addition to the off-target effects and the immune response described above, ethical issues are also of concern for this technique, which have long been debated. Despite the limitations of the applications of CRISPR-Cas9 gene-editing technology, we believe that CRISPR-Cas9 gene-editing technology will contribute to improving the treatment of cancer.

## Data Availability

Not applicable for this study.

## Abbreviations

CRISPR: Clustered regularly interspaced short palindromic repeats
Cas9: CRISPR-associated protein 9
SgRNA: Single guide RNA
PAM: Protospacer-adjacent motif
NHEJ: Nonhomologous end-joining
HDR: Homology-directed repair
DSBs: Double-strand breaks

## References

[1] Montaño A, Forero-Castro M, Hernández-Rivas JM, García-Tuñón I, Benito R (2018). Targeted genome editing in acute lymphoblastic leukemia: a review. BMC Biotechnol.

[2] Stupp R, Hegi ME, Mason WP, van den Bent MJ, Taphoorn MJ, Janzer RC, Ludwin SK, Allgeier A, Fisher B, Belanger K (2009). Effects of radiotherapy with concomitant and adjuvant temozolomide versus radiotherapy alone on survival in glioblastoma in a randomised phase III study: 5-year analysis of the EORTC-NCIC trial. Lancet Oncol.

[3] Liu C, Zhang L, Liu H, Cheng K (2017). Delivery strategies of the CRISPR-Cas9 gene-editing system for therapeutic applications. J Control Release.

[4] White MK, Khalili K (2016). CRISPR/Cas9 and cancer targets: future possibilities and present challenges. Oncotarget.

[5] Fellmann C, Gowen BG, Lin PC, Doudna JA, Corn JE (2017). Cornerstones of CRISPR-Cas in drug discovery and therapy. Nat Rev Drug Discov.

[6] Cyranoski D (2016). CRISPR gene-editing tested in a person for the first time. Nature.

[7] Fire A, Xu S, Montgomery MK, Kostas SA, Driver SE, Mello CC (1998). Potent and specific genetic interference by double-stranded RNA in Caenorhabditis elegans. Nature.

[8] Sontheimer EJ (2005). Assembly and function of RNA silencing complexes. Nat Rev Mol Cell Biol.

[9] Urnov FD, Rebar EJ, Holmes MC, Zhang HS, Gregory PD (2010). Genome editing with engineered zinc finger nucleases. Nat Rev Genet.

[10] Christian M, Cermak T, Doyle EL, Schmidt C, Zhang F, Hummel A, Bogdanove AJ, Voytas DF (2010). Targeting DNA double-strand breaks with TAL effector nucleases. Genetics.

[11] Waryah CB, Moses C, Arooj M, Blancafort P (2018). Zinc fingers, TALEs, and CRISPR systems: a comparison of tools for epigenome editing. Methods Mol Biol.

[12] Ishino Y, Shinagawa H, Makino K, Amemura M, Nakata A (1987). Nucleotide sequence of the iap gene, responsible for alkaline phosphatase isozyme conversion in Escherichia coli, and identification of the gene product. J Bacteriol.

[13] Makarova KS, Wolf YI, Alkhnbashi OS, Costa F, Shah SA, Saunders SJ, Barrangou R, Brouns SJ, Charpentier E, Haft DH (2015). An updated evolutionary classification of CRISPR-Cas systems. Nat Rev Microbiol.

[14] Cong L, Ran FA, Cox D, Lin S, Barretto R, Habib N, Hsu PD, Wu X, Jiang W, Marraffini LA (2013). Multiplex genome engineering using CRISPR/Cas systems. Science.

[15] Mali P, Yang L, Esvelt KM, Aach J, Guell M, DiCarlo JE, Norville JE, Church GM (2013). RNA-guided human genome engineering via Cas9. Science.

[16] Jinek M, Chylinski K, Fonfara I, Hauer M, Doudna JA, Charpentier E (2012). A programmable dual-RNA-guided DNA endonuclease in adaptive bacterial immunity. Science.

[17] Bolotin A, Quinquis B, Sorokin A, Ehrlich SD (2005). Clustered regularly interspaced short palindrome repeats (CRISPRs) have spacers of extrachromosomal origin. Microbiology (Reading).

[18] Hsu PD, Scott DA, Weinstein JA, Ran FA, Konermann S, Agarwala V, Li Y, Fine EJ, Wu X, Shalem O (2013). DNA targeting specificity of RNA-guided Cas9 nucleases. Nat Biotechnol.

[19] Malina A, Mills JR, Cencic R, Yan Y, Fraser J, Schippers LM, Paquet M, Dostie J, Pelletier J (2013). Repurposing CRISPR/Cas9 for in situ functional assays. Genes Dev.

[20] Picco G, Chen ED, Alonso LG, Behan FM, Gonçalves E, Bignell G, Matchan A, Fu B, Banerjee R, Anderson E (2019). Functional linkage of gene fusions to cancer cell fitness assessed by pharmacological and CRISPR-Cas9 screening. Nat Commun.

[21] Koike-Yusa H, Li Y, Tan EP, Velasco-Herrera Mdel C, Yusa K (2014). Genome-wide recessive genetic screening in mammalian cells with a lentiviral CRISPR-guide RNA library. Nat Biotechnol.

[22] Lin ML, Park JH, Nishidate T, Nakamura Y, Katagiri T (2007). Involvement of maternal embryonic leucine zipper kinase (MELK) in mammary carcinogenesis through interaction with Bcl-G, a pro-apoptotic member of the Bcl-2 family. Breast Cancer Res.

[23] Gray D, Jubb AM, Hogue D, Dowd P, Kljavin N, Yi S, Bai W, Frantz G, Zhang Z, Koeppen H (2005). Maternal embryonic leucine zipper kinase/murine protein serine-threonine kinase 38 is a promising therapeutic target for multiple cancers. Cancer Res.

[24] Kuner R, Fälth M, Pressinotti NC, Brase JC, Puig SB, Metzger J, Gade S, Schäfer G, Bartsch G, Steiner E (2013). The maternal embryonic leucine zipper kinase (MELK) is upregulated in high-grade prostate cancer. J Mol Med (Berl).

[25] Lin A, Giuliano CJ, Sayles NM, Sheltzer JM: CRISPR/Cas9 mutagenesis invalidates a putative cancer dependency targeted in on-going clinical trials. Elife. 2017;6:e24179. 10.7554/eLife.24179 PMC5365317 28337968

[26] Kodama M, Kodama T, Newberg JY, Katayama H, Kobayashi M, Hanash SM, Yoshihara K, Wei Z, Tien JC, Rangel R (2017). In vivo loss-of-function screens identify KPNB1 as a new druggable oncogene in epithelial ovarian cancer. Proc Natl Acad Sci U S A.

[27] Meyers RM, Bryan JG, McFarland JM, Weir BA, Sizemore AE, Xu H, Dharia NV, Montgomery PG, Cowley GS, Pantel S (2017). Computational correction of copy number effect improves specificity of CRISPR-Cas9 essentiality screens in cancer cells. Nat Genet.

[28] Behan FM, Iorio F, Picco G, Gonçalves E, Beaver CM, Migliardi G, Santos R, Rao Y, Sassi F, Pinnelli M (2019). Prioritization of cancer therapeutic targets using CRISPR-Cas9 screens. Nature.

[29] Wang T, Yu H, Hughes NW, Liu B, Kendirli A, Klein K, Chen WW, Lander ES, Sabatini DM (2017). Gene essentiality profiling reveals gene networks and synthetic lethal interactions with oncogenic ras. Cell.

[30] Chen S, Sanjana NE, Zheng K, Shalem O, Lee K, Shi X, Scott DA, Song J, Pan JQ, Weissleder R (2015). Genome-wide CRISPR screen in a mouse model of tumor growth and metastasis. Cell.

[31] Chow RD, Guzman CD, Wang G, Schmidt F, Youngblood MW, Ye L, Errami Y, Dong MB, Martinez MA, Zhang S (2017). AAV-mediated direct in vivo CRISPR screen identifies functional suppressors in glioblastoma. Nat Neurosci.

[32] Shalem O, Sanjana NE, Hartenian E, Shi X, Scott DA, Mikkelson T, Heckl D, Ebert BL, Root DE, Doench JG (2014). Genome-scale CRISPR-Cas9 knockout screening in human cells. Science.

[33] Doench JG, Hartenian E, Graham DB, Tothova Z, Hegde M, Smith I, Sullender M, Ebert BL, Xavier RJ, Root DE (2014). Rational design of highly active sgRNAs for CRISPR-Cas9-mediated gene inactivation. Nat Biotechnol.

[34] Shi J, Wang E, Milazzo JP, Wang Z, Kinney JB, Vakoc CR (2015). Discovery of cancer drug targets by CRISPR-Cas9 screening of protein domains. Nat Biotechnol.

[35] Najm FJ, Strand C, Donovan KF, Hegde M, Sanson KR, Vaimberg EW, Sullender ME, Hartenian E, Kalani Z, Fusi N (2018). Orthologous CRISPR-Cas9 enzymes for combinatorial genetic screens. Nat Biotechnol.

[36] Aguirre AJ, Meyers RM, Weir BA, Vazquez F, Zhang CZ, Ben-David U, Cook A, Ha G, Harrington WF, Doshi MB (2016). Genomic copy number dictates a gene-independent cell response to CRISPR/Cas9 targeting. Cancer Discov.

[37] Cao J, Wei J, Yang P, Zhang T, Chen Z, He F, Wei F, Chen H, Hu H, Zhong J (2018). Genome-scale CRISPR-Cas9 knockout screening in gastrointestinal stromal tumor with Imatinib resistance. Mol Cancer.

[38] Wei L, Lee D, Law CT, Zhang MS, Shen J, Chin DW, Zhang A, Tsang FH, Wong CL, Ng IO (2019). Genome-wide CRISPR/Cas9 library screening identified PHGDH as a critical driver for Sorafenib resistance in HCC. Nat Commun.

[39] Shin HY, Wang C, Lee HK, Yoo KH, Zeng X, Kuhns T, Yang CM, Mohr T, Liu C, Hennighausen L (2017). CRISPR/Cas9 targeting events cause complex deletions and insertions at 17 sites in the mouse genome. Nat Commun.

[40] Kosicki M, Tomberg K, Bradley A (2018). Repair of double-strand breaks induced by CRISPR-Cas9 leads to large deletions and complex rearrangements. Nat Biotechnol.

[41] Qi LS, Larson MH, Gilbert LA, Doudna JA, Weissman JS, Arkin AP, Lim WA (2013). Repurposing CRISPR as an RNA-guided platform for sequence-specific control of gene expression. Cell.

[42] Liu SJ, Malatesta M, Lien BV, Saha P, Thombare SS, Hong SJ, Pedraza L, Koontz M, Seo K, Horlbeck MA (2020). CRISPRi-based radiation modifier screen identifies long non-coding RNA therapeutic targets in glioma. Genome Biol.

[43] Liu S, Harmston N, Glaser TL, Wong Y, Zhong Z, Madan B, Virshup DM, Petretto E (2020). Wnt-regulated lncRNA discovery enhanced by in vivo identification and CRISPRi functional validation. Genome Med.

[44] Bikard D, Jiang W, Samai P, Hochschild A, Zhang F, Marraffini LA (2013). Programmable repression and activation of bacterial gene expression using an engineered CRISPR-Cas system. Nucleic Acids Res.

[45] Gilbert LA, Larson MH, Morsut L, Liu Z, Brar GA, Torres SE, Stern-Ginossar N, Brandman O, Whitehead EH, Doudna JA (2013). CRISPR-mediated modular RNA-guided regulation of transcription in eukaryotes. Cell.

[46] Ramos NR, Mo CC, Karp JE, Hourigan CS (2015). Current approaches in the treatment of relapsed and refractory acute myeloid leukemia. J Clin Med.

[47] Bester AC, Lee JD, Chavez A, Lee YR, Nachmani D, Vora S, Victor J, Sauvageau M, Monteleone E, Rinn JL (2018). An integrated genome-wide CRISPRa approach to functionalize lncRNAs in drug resistance. Cell.

[48] Gaj T, Epstein BE, Schaffer DV (2016). Genome engineering using adeno-associated virus: basic and clinical research applications. Mol Ther.

[49] Paulk NK, Wursthorn K, Wang Z, Finegold MJ, Kay MA, Grompe M (2010). Adeno-associated virus gene repair corrects a mouse model of hereditary tyrosinemia in vivo. Hepatology.

[50] Wang G, Chow RD, Ye L, Guzman CD, Dai X, Dong MB, Zhang F, Sharp PA, Platt RJ, Chen S: Mapping a functional cancer genome atlas of tumor suppressors in mouse liver using AAV-CRISPR-mediated direct in vivo screening. Sci Adv. 2018;4(2):eaao5508. 10.1126/sciadv.aao5508 PMC5829971 29503867

[51] Wang C, Wang G, Feng X, Shepherd P, Zhang J, Tang M, Chen Z, Srivastava M, McLaughlin ME, Navone NM (2019). Genome-wide CRISPR screens reveal synthetic lethality of RNASEH2 deficiency and ATR inhibition. Oncogene.

[52] Wu Z, Yang H, Colosi P (2010). Effect of genome size on AAV vector packaging. Mol Ther.

[53] Kumar M, Keller B, Makalou N, Sutton RE (2001). Systematic determination of the packaging limit of lentiviral vectors. Hum Gene Ther.

[54] Platt RJ, Chen S, Zhou Y, Yim MJ, Swiech L, Kempton HR, Dahlman JE, Parnas O, Eisenhaure TM, Jovanovic M (2014). CRISPR-Cas9 knockin mice for genome editing and cancer modeling. Cell.

[55] Ding Q, Strong A, Patel KM, Ng SL, Gosis BS, Regan SN, Cowan CA, Rader DJ, Musunuru K (2014). Permanent alteration of PCSK9 with in vivo CRISPR-Cas9 genome editing. Circ Res.

[56] Maddalo D, Manchado E, Concepcion CP, Bonetti C, Vidigal JA, Han YC, Ogrodowski P, Crippa A, Rekhtman N, de Stanchina E (2014). In vivo engineering of oncogenic chromosomal rearrangements with the CRISPR/Cas9 system. Nature.

[57] Schirmbeck R, Reimann J, Kochanek S, Kreppel F (2008). The immunogenicity of adenovirus vectors limits the multispecificity of CD8 T-cell responses to vector-encoded transgenic antigens. Mol Ther.

[58] Chen Z, Liu F, Chen Y, Liu J, Wang X, Chen AT, Deng G, Zhang H, Liu J, Hong Z et al: Targeted Delivery of CRISPR/Cas9-Mediated Cancer Gene Therapy via Liposome-Templated Hydrogel Nanoparticles. Adv Funct Mater. 2017;27(46):1703036. 10.1002/adfm.201703036 PMC5939593 29755309

[59] Xu C, Qi X, Du X, Zou H, Gao F, Feng T, Lu H, Li S, An X, Zhang L (2017). piggyBac mediates efficient in vivo CRISPR library screening for tumorigenesis in mice. Proc Natl Acad Sci USA.

[60] Rouet R, Thuma BA, Roy MD, Lintner NG, Rubitski DM, Finley JE, Wisniewska HM, Mendonsa R, Hirsh A, de Oñate L (2018). Receptor-mediated delivery of CRISPR-Cas9 endonuclease for cell-type-specific gene editing. J Am Chem Soc.

[61] Lawrence MS, Stojanov P, Mermel CH, Robinson JT, Garraway LA, Golub TR, Meyerson M, Gabriel SB, Lander ES, Getz G (2014). Discovery and saturation analysis of cancer genes across 21 tumour types. Nature.

[62] Xue W, Chen S, Yin H, Tammela T, Papagiannakopoulos T, Joshi NS, Cai W, Yang G, Bronson R, Crowley DG (2014). CRISPR-mediated direct mutation of cancer genes in the mouse liver. Nature.

[63] Weber J, Öllinger R, Friedrich M, Ehmer U, Barenboim M, Steiger K, Heid I, Mueller S, Maresch R, Engleitner T (2015). CRISPR/Cas9 somatic multiplex-mutagenesis for high-throughput functional cancer genomics in mice. Proc Natl Acad Sci USA.

[64] Rahib L, Smith BD, Aizenberg R, Rosenzweig AB, Fleshman JM, Matrisian LM (2014). Projecting cancer incidence and deaths to 2030: the unexpected burden of thyroid, liver, and pancreas cancers in the United States. Cancer Res.

[65] Maresch R, Mueller S, Veltkamp C, Öllinger R, Friedrich M, Heid I, Steiger K, Weber J, Engleitner T, Barenboim M (2016). Multiplexed pancreatic genome engineering and cancer induction by transfection-based CRISPR/Cas9 delivery in mice. Nat Commun.

[66] Chiou SH, Winters IP, Wang J, Naranjo S, Dudgeon C, Tamburini FB, Brady JJ, Yang D, Grüner BM, Chuang CH (2015). Pancreatic cancer modeling using retrograde viral vector delivery and in vivo CRISPR/Cas9-mediated somatic genome editing. Genes Dev.

[67] Siegel RL, Miller KD, Fedewa SA, Ahnen DJ, Meester RGS, Barzi A, Jemal A (2017). Colorectal cancer statistics, 2017. CA Cancer J Clin.

[68] Drost J, van Boxtel R, Blokzijl F, Mizutani T, Sasaki N, Sasselli V, de Ligt J, Behjati S, Grolleman JE, van Wezel T (2017). Use of CRISPR-modified human stem cell organoids to study the origin of mutational signatures in cancer. Science.

[69] de Sousa e Melo F, Kurtova AV, Harnoss JM, Kljavin N, Hoeck JD, Hung J, Anderson J, Storm EE, Modrusan Z, Koeppen H (2017). A distinct role for Lgr5(+) stem cells in primary and metastatic colon cancer. Nature.

[70] O’Rourke KP, Loizou E, Livshits G, Schatoff EM, Baslan T, Manchado E, Simon J, Romesser PB, Leach B, Han T, et al. Transplantation of engineered organoids enables rapid generation of metastatic mouse models of colorectal cancer. Nat Biotechnol. 2017;35(6):577–82. 10.1038/nbt.3837 PMC5462850 28459450

[71] Roper J, Tammela T, Akkad A, Almeqdadi M, Santos SB, Jacks T, Yilmaz ÖH (2018). Colonoscopy-based colorectal cancer modeling in mice with CRISPR-Cas9 genome editing and organoid transplantation. Nat Protoc.

[72] Grzywa-Celińska A, Drogoń I, Emeryk-Maksymiuk J, Chmielewska I, Milanowski J (2019). Not only cigarettes - other culprits of lung cancer. Ann Agric Environ Med.

[73] DeSantis CE, Ma J, Gaudet MM, Newman LA, Miller KD, Goding Sauer A, Jemal A, Siegel RL (2019). Breast cancer statistics, 2019. CA Cancer J Clin.

[74] Annunziato S, Lutz C, Henneman L, Bhin J, Wong K, Siteur B, van Gerwen B, de Korte-Grimmerink R, Zafra MP, Schatoff EM (2020). In situ CRISPR-Cas9 base editing for the development of genetically engineered mouse models of breast cancer. Embo j.

[75] Dekkers JF, Whittle JR, Vaillant F, Chen HR, Dawson C, Liu K, Geurts MH, Herold MJ, Clevers H, Lindeman GJ (2020). Modeling breast cancer using CRISPR-Cas9-mediated engineering of human breast organoids. J Natl Cancer Inst.

[76] Koo T, Yoon AR, Cho HY, Bae S, Yun CO, Kim JS (2017). Selective disruption of an oncogenic mutant allele by CRISPR/Cas9 induces efficient tumor regression. Nucleic Acids Res.

[77] Chen ZH, Yu YP, Zuo ZH, Nelson JB, Michalopoulos GK, Monga S, Liu S, Tseng G, Luo JH (2017). Targeting genomic rearrangements in tumor cells through Cas9-mediated insertion of a suicide gene. Nat Biotechnol.

[78] Tsai SQ, Zheng Z, Nguyen NT, Liebers M, Topkar VV, Thapar V, Wyvekens N, Khayter C, Iafrate AJ, Le LP (2015). GUIDE-seq enables genome-wide profiling of off-target cleavage by CRISPR-Cas nucleases. Nat Biotechnol.

[79] Zhang L, Jia R, Palange NJ, Satheka AC, Togo J, An Y, Humphrey M, Ban L, Ji Y, Jin H (2015). Large genomic fragment deletions and insertions in mouse using CRISPR/Cas9. PLoS ONE.

[80] Gaudelli NM, Komor AC, Rees HA, Packer MS, Badran AH, Bryson DI, Liu DR (2017). Programmable base editing of A•T to G•C in genomic DNA without DNA cleavage. Nature.

[81] Komor AC, Kim YB, Packer MS, Zuris JA, Liu DR (2016). Programmable editing of a target base in genomic DNA without double-stranded DNA cleavage. Nature.

[82] Pardoll DM (2012). The blockade of immune checkpoints in cancer immunotherapy. Nat Rev Cancer.

[83] Berger R, Rotem-Yehudar R, Slama G, Landes S, Kneller A, Leiba M, Koren-Michowitz M, Shimoni A, Nagler A (2008). Phase I safety and pharmacokinetic study of CT-011, a humanized antibody interacting with PD-1, in patients with advanced hematologic malignancies. Clin Cancer Res.

[84] Taube JM, Klein A, Brahmer JR, Xu H, Pan X, Kim JH, Chen L, Pardoll DM, Topalian SL, Anders RA (2014). Association of PD-1, PD-1 ligands, and other features of the tumor immune microenvironment with response to anti-PD-1 therapy. Clin Cancer Res.

[85] Manguso RT, Pope HW, Zimmer MD, Brown FD, Yates KB, Miller BC, Collins NB, Bi K, LaFleur MW, Juneja VR (2017). In vivo CRISPR screening identifies Ptpn2 as a cancer immunotherapy target. Nature.

[86] Park JH, Geyer MB, Brentjens RJ (2016). CD19-targeted CAR T-cell therapeutics for hematologic malignancies: interpreting clinical outcomes to date. Blood.

[87] Louis CU, Savoldo B, Dotti G, Pule M, Yvon E, Myers GD, Rossig C, Russell HV, Diouf O, Liu E (2011). Antitumor activity and long-term fate of chimeric antigen receptor-positive T cells in patients with neuroblastoma. Blood.

[88] Eyquem J, Mansilla-Soto J, Giavridis T, van der Stegen SJ, Hamieh M, Cunanan KM, Odak A, Gönen M, Sadelain M (2017). Targeting a CAR to the TRAC locus with CRISPR/Cas9 enhances tumour rejection. Nature.

[89] Ren J, Liu X, Fang C, Jiang S, June CH, Zhao Y (2017). Multiplex genome editing to generate universal CAR T cells resistant to PD1 inhibition. Clin Cancer Res.

[90] Duan Y, Wang Z, Xu L, Sun L, Song H, Yin H, He F (2020). lncRNA SNHG3 acts as a novel tumor suppressor and regulates tumor proliferation and metastasis via AKT/mTOR/ERK pathway in papillary thyroid carcinoma. J Cancer.

[91] Wang X, Yu B, Jin Q, Zhang J, Yan B, Yang L, Li Y, Li Q, Wang P, Sun C (2020). Regulation of laryngeal squamous cell cancer progression by the lncRNA RP11–159K7.2/miR-206/DNMT3A axis. J Cell Mol Med.

[92] Bai L, Wang H, Wang AH, Zhang LY, Bai J (2017). MicroRNA-532 and microRNA-3064 inhibit cell proliferation and invasion by acting as direct regulators of human telomerase reverse transcriptase in ovarian cancer. PLoS ONE.

[93] Yan J, Jia Y, Chen H, Chen W, Zhou X (2019). Long non-coding RNA PXN-AS1 suppresses pancreatic cancer progression by acting as a competing endogenous RNA of miR-3064 to upregulate PIP4K2B expression. J Exp Clin Cancer Res.

[94] Fu Y, Foden JA, Khayter C, Maeder ML, Reyon D, Joung JK, Sander JD (2013). High-frequency off-target mutagenesis induced by CRISPR-Cas nucleases in human cells. Nat Biotechnol.

[95] Lin Y, Cradick TJ, Brown MT, Deshmukh H, Ranjan P, Sarode N, Wile BM, Vertino PM, Stewart FJ, Bao G (2014). CRISPR/Cas9 systems have off-target activity with insertions or deletions between target DNA and guide RNA sequences. Nucleic Acids Res.

[96] Lazzarotto CR, Malinin NL, Li Y, Zhang R, Yang Y, Lee G, Cowley E, He Y, Lan X, Jividen K (2020). CHANGE-seq reveals genetic and epigenetic effects on CRISPR-Cas9 genome-wide activity. Nat Biotechnol.

[97] Tsai SQ, Nguyen NT, Malagon-Lopez J, Topkar VV, Aryee MJ, Joung JK (2017). CIRCLE-seq: a highly sensitive in vitro screen for genome-wide CRISPR-Cas9 nuclease off-targets. Nat Methods.

[98] Lazzarotto CR, Nguyen NT, Tang X, Malagon-Lopez J, Guo JA, Aryee MJ, Joung JK, Tsai SQ (2018). Defining CRISPR-Cas9 genome-wide nuclease activities with CIRCLE-seq. Nat Protoc.

[99] Akcakaya P, Bobbin ML, Guo JA, Malagon-Lopez J, Clement K, Garcia SP, Fellows MD, Porritt MJ, Firth MA, Carreras A (2018). In vivo CRISPR editing with no detectable genome-wide off-target mutations. Nature.

[100] Doench JG, Fusi N, Sullender M, Hegde M, Vaimberg EW, Donovan KF, Smith I, Tothova Z, Wilen C, Orchard R (2016). Optimized sgRNA design to maximize activity and minimize off-target effects of CRISPR-Cas9. Nat Biotechnol.

[101] Kleinstiver BP, Pattanayak V, Prew MS, Tsai SQ, Nguyen NT, Zheng Z, Joung JK (2016). High-fidelity CRISPR-Cas9 nucleases with no detectable genome-wide off-target effects. Nature.

[102] Wang D, Mou H, Li S, Li Y, Hough S, Tran K, Li J, Yin H, Anderson DG, Sontheimer EJ (2015). Adenovirus-mediated somatic genome editing of Pten by CRISPR/Cas9 in mouse liver in spite of Cas9-specific immune responses. Hum Gene Ther.

[103] Chew WL, Tabebordbar M, Cheng JK, Mali P, Wu EY, Ng AH, Zhu K, Wagers AJ, Church GM (2016). A multifunctional AAV-CRISPR-Cas9 and its host response. Nat Methods.

[104] Ajina R, Zamalin D, Zuo A, Moussa M, Catalfamo M, Jablonski SA, Weiner LM (2019). SpCas9-expression by tumor cells can cause T cell-dependent tumor rejection in immunocompetent mice. Oncoimmunology.

